# egfr overexpression in squamous cell carcinoma of the penis

**DOI:** 10.3747/co.v17i1.471

**Published:** 2010-02

**Authors:** N. Lavens, R. Gupta, L.A. Wood

**Affiliations:** * Urology Department, QEII Health Sciences Centre, Halifax, NS; † Department of Anatomical Pathology and Laboratory Medicine, QEII Health Sciences Centre, Halifax, NS; ‡ Division of Medical Oncology, QEII Health Sciences Centre, Halifax, NS

**Keywords:** egfr overexpression, penile cancer

## 1. INTRODUCTION

Penile cancer is a rare cancer in developed countries, but it has a higher incidence in developing countries such as Asia, Africa, and South America 1,2. Despite its rarity, the disease significantly affects the patient’s life when it does occur. Localized disease can be managed by surgical resection (partial or total penectomy) or penis-preserving surgery and radiotherapy. In patients with a higher T stage or grade, or clinically positive inguinal lymph nodes, an inguinal lymph node dissection is usually performed. If nonregional lymph nodes are involved or if metastatic disease is present, surgical cure cannot be obtained.

In these cases of advanced disease, surgery with or without radiation is often used for palliation and local control. Metastatic disease, which can involve distant lymph nodes, lung, liver, brain, and bone, is often managed with chemotherapy. Chemotherapy in this situation has been poorly studied (no randomized trials), responses are often partial and of short duration, and therapy is associated with significant toxicity 3–7. Thus, it is imperative that new therapies be investigated for this rare but devastating disease.

Since the end of the 1980s, an understanding of the molecular biology of cancer has immensely increased the understanding of tumour biology. Using this knowledge, scientists and clinicians are turning to novel treatments to exploit specific molecular targets. Tumour growth and progression depend on a number of pathways involving receptors at the cell membrane surface that control intracellular signalling pathways. One important pathway involves the cell-surface receptor epidermal growth factor receptor (egfr) 8. Epidermal growth factor and other ligands bind to and activate egfr to activate signalling pathways that regulate cell proliferation, migration, adhesion, differentiation, and apoptosis.

In a number of solid tumours, including brain, lung, breast, prostate, stomach, and head and neck, egfr can be overexpressed or mutated, leading to deregulation of related pathways. Overstimulation of egfr-mediated signalling can contribute to uncontrolled cell division and thus to oncogenic signalling and tumour angiogenesis and metastasis, protecting cancer cells from undergoing apoptosis. In some malignancies, overexpression or excessive activation of egfr tends to be associated with a more aggressive malignant phenotype, with greater metastatic potential and worse prognosis 9–11.

To our knowledge, no studies have evaluated the expression of egfr in invasive squamous cell carcinoma of the penis. Given egfr overexpression, and the success with egfr-directed therapy in other tumours, we felt it worthwhile to pursue this area of research in penile cancer. Thus, the objective of the present study was to characterize the expression of egfr in cases of invasive squamous cell carcinoma of the penis. If overexpression were to be seen, then exploiting the egfr pathway as a therapeutic target in advanced penile cancer would be a rational approach.

## 2. MATERIALS AND METHODS

Approval from the local research ethics board was obtained. Samples were selected from a pathology database of invasive penile squamous cell carcinoma obtained through biopsy or surgical resection at the QEII Health Sciences Center, Halifax, during 1997–2004.

Expression of egfr was evaluated and graded (0, 1+, 2+, 3+) by one trained pathologist using guidelines outlined in the commercially available immunohistochemical system kit EGFR PharmDx (DakoCytomation, Carpinteria, CA, U.S.A.). We used the EGFR PharmDx monoclonal mouse antibody (clone 2-18C9) with controls consisting of human cell lines CAMA-1 (negative control) and HT29 (positive control). Overexpression of egfr in the primary penile cancer was defined as any immunohistologic staining of tumour cell membranes above background level, whether circumferential staining was complete or incomplete. No information on lymph node staging, lymphadenectomies, or egfr expression in lymph nodes was obtained.

## 3. RESULTS

From the selected 8-year time period, 17 cases were obtained and studied. The median age of all patients was 66 years (range: 46–95 years).

**FIGURE 1:**
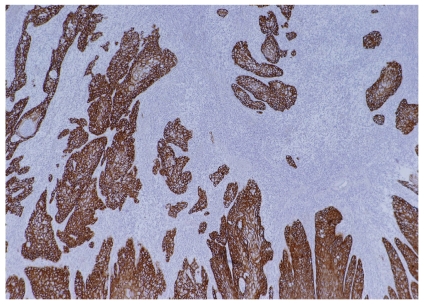
Example of overexpression of epidermal growth factor receptor in invasive squamous cell carcinoma of the penis.

All 17 cases overexpressed egfr (Table I, Figure 1). Expression was 3+ in 14 cases and 2+ in 3 cases. Of the samples examined, 4 cases were also associated with carcinoma *in situ* (cis). In 2 cases, the cis also overexpressed egfr, but to a lesser degree (1+) than did the invasive component. In the other 2 cases, the cis did not overexpress egfr.

In all cases, the adjacent normal squamous epithelium demonstrated normal background egfr expression. Table I also presents the pathologic T stage and grade, but because overexpression of egfr was seen in all samples, no associations can be made between stage, grade, and overexpression.

Limitations to this study include its small sample size, which limited our ability to make any comments or conclusions about associations with T stage and grade. Also, we did not obtain information on lymph node metastases or measure the phosphorylated form of egfr.

## 4. DISCUSSION AND CONCLUSIONS

Therapeutic advances in the area of advanced or metastatic penile cancer have been limited for several decades. Managing patients with this disease is a frustrating and disappointing endeavour because only short-lived partial responses can be obtained using traditional chemotherapy drugs.

Every sample of invasive squamous cell carcinoma of the penis evaluated in this study expressed egfr, with most showing 3+ overexpression. To date, several egfr-targeted therapies have been developed. These include monoclonal antibodies that bind to egfr ligands (for example, cetuximab) and egfr tyrosine kinase inhibitors (for example, gefitinib, erlotinib). As single agents, these drugs have been shown to have activity in several solid tumours including lung, head and neck, and colon 12–16. In phase iii lung and colon cancer trials, overall survival was improved. Current research is ongoing in these tumours to study the effects of chemotherapy in combination with egfr-targeted therapy to improve outcomes even more.

Given the positive results in other tumours, the high degree of egfr overexpression in all samples in this study, and the lack of effective treatment for advanced penile cancer, further research into the egfr pathway and invasive penile cancer are warranted. For example, determining whether lymph node or distant metastases from penile cancer also overexpress egfr would be worthwhile, as would determining whether egfr-targeted therapy has clinical activity in the setting of advanced disease.

## Figures and Tables

**TABLE I:** Expression of epidermal growth factor receptor (egfr) in invasive squamous cell carcinoma of the penis

Sample	Primary tumour		egfr expression	Associated carcinoma *in situ*
	Stage	Grade		
1	Not available		3+	—
2	Not available		3+	—
3	T1	1	2+	—
4	T1	1	2+	—
5	T1	1	3+	—
6	T1	1	3+	0
7	T1	1	3+	—
8	T1	2	3+	—
9	T1	2	3+	—
10	T1	2	3+	—
11	T1	3	2+	—
12	T1	3	3+	1+
13	T2	2	3+	—
14	T2	2	3+	0
15	T2	2	3+	—
16	T3	2	3+	—
17	T3	3	3+	1+
